# Primary melanoma of leptomeninge in a patient with giant congenital melanocytic nevus^☆^^☆☆^

**DOI:** 10.1016/j.abd.2019.11.002

**Published:** 2020-03-18

**Authors:** Adriana Kamilly Leitão Pitman Machado, Débora Bacellar Cruz Nunes, Francisca Regina Oliveira Carneiro, Alena Margareth Darwich Mendes

**Affiliations:** Service of Dermatology, Universidade do Estado do Pará, Belém, PA, Brazil

Dear Editor,

Congenital Melanocytic Nevus (CMN) is characterized by pigmented lesions present at birth or in the first weeks of life. The Giant Congenital Melanocytic Nevus (GCMN) form presents a minimum extension of 20 cm in adult life and is rare (1:20,000 newborns). In addition to being an unsightly condition, giant CMN presents additional risks of extracutaneous morbidities such as neurological complications. A complicating factor is the presence of melanocytic cells in the Central Nervous System (CNS), a comorbidity called Neurocutaneous Melanosis (NCM).^1^

We report a case of female patient, accompanied since the age of 2 years with GCMN in garment. In clinical follow-up, no neurological symptoms or changes in psychomotor development were detected. A brain Magnetic Resonance Imaging (MRI) confirmed the presence of T1-Weighted (W) images in the left parietal region, corresponding to neurocutaneous melanosis. The diagnosis was made upon typical imaging and skin findings.

Dermatological examination revealed a brownish-black plaque, with evident hypertrichosis, extending from the cervical region to the knees, garment-like, with multiple satellite lesions on the face, upper and lower limbs (Fig. 1). The patient had nodules on the back corresponding to schawannoma. At 10 years old, after loss of clinical follow-up for 3 years, she started a sudden onset of seizures, right hemisphere paresis, headache and vomiting.

**Figure 1:**
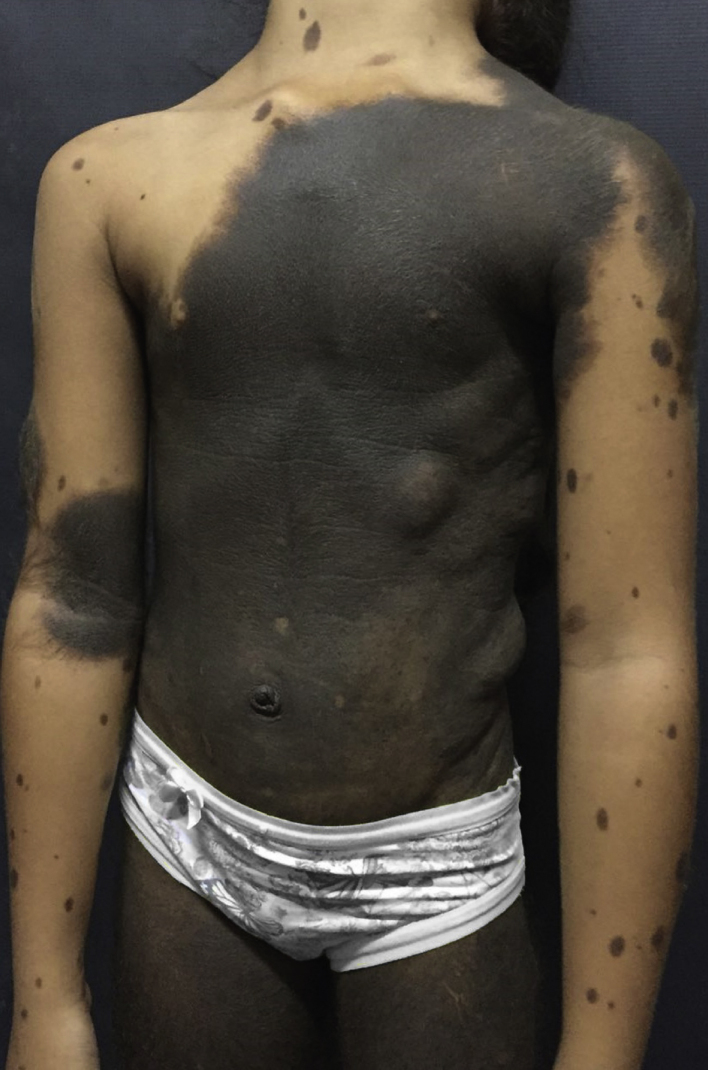
Giant congenital melanocytic nevus in garment with multiple satellite lesions.

Brain MRI scan demonstrated the presence of a single solid expansive lesion measuring 5 cm × 3.5 cm in the left fronto-parietal region, associated with an intense vasogenic edema, promoting midline deviation (Fig. 2). Histopathology showed a neoplasm formed by the proliferation of atypical cells, containing granular brown pigment similar to melanin and with hyperchromatic, enlarged central nuclei with evident nucleoli, frequent atypical mitoses, preferentially infiltrating the meningeal but also the adjacent brain parenchyma, amid areas of necrosis and hemorrhage (Fig. 3).

**Figure 2:**
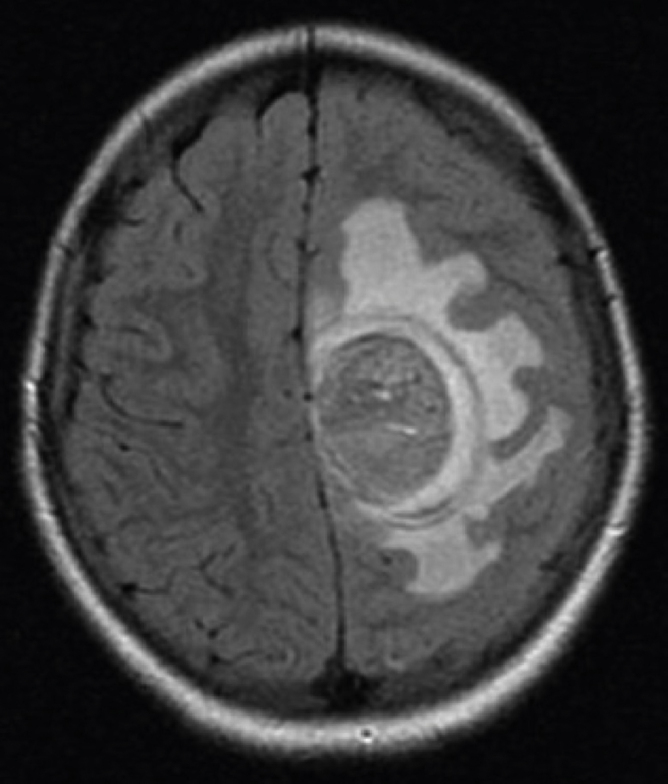
Presence of a single solid expansive lesion measuring 5 cm × 3.5 cm in the left fronto-parietal region, associated with an intense vasogenic edema, promoting midline deviation.

**Figure 3:**
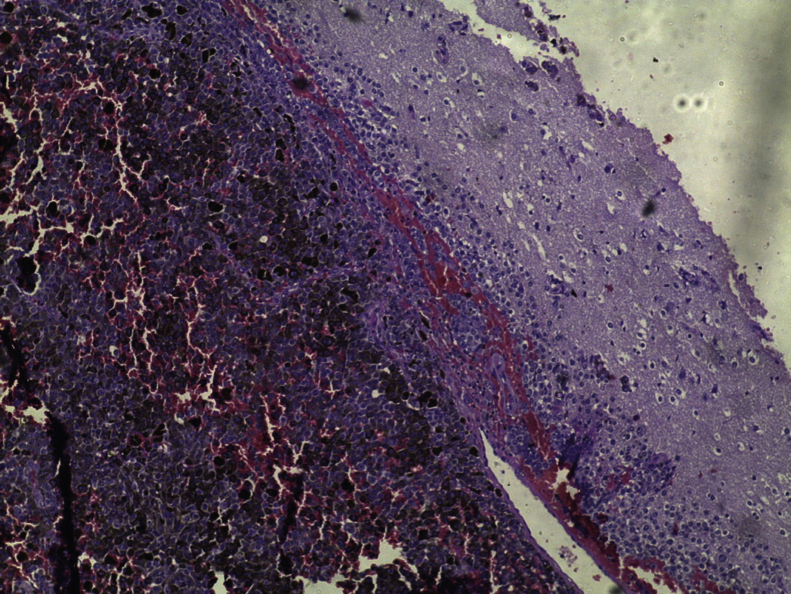
Neoplasm formed by the proliferation of atypical cells containing pigment preferentially infiltrating the meningeal but also the adjacent brain parenchyma, amid areas of necrosis and hemorrhage (Hematoxylin & eosin, ×40).

The immunohistochemical examination showed strong reactivity to the panel of antibodies S100, HBM45 and Melan A. Additional imaging studies showed no metastasis. The final diagnosis was primary melanoma of leptomeningeal. The patient died from intracranial hemorrhage followed by cardiorespiratory arrest four months after diagnosis.

Primary CNS melanoma is a rare disease. It represents 1% of melanomas and approximately 0.05% of primary malignancies of cranial tumors. These can be divided into nodular intraparenchymal and diffuse leptomeningeal patterns.^2^ Primary leptomeningel Malignant Melanoma (MM) is extremely rare, with an incidence of one case per 20 million individuals, generally showing aggressive progression and resistance to chemotherapy and radiotherapy.^1^, ^2^

The risk of estimated lifetime MM-all sites for individuals with CMN is around 5%, with increased risk to 12% in patients with neurocutaneous melanosis. This is characterized by the migration and erroneous proliferation of melanocytic cells in the CNS from neural crest melanoblasts.^2^, ^3^

NCM involves several additional comorbidities which include hydrocephalus, convulsions, cranial nerve palsy, neuropsychiatric disorders and the risk of malignant degeneration of the cells. Mortality rate is close to 100% for CNS MM cases and 70% of patients with neurocutaneous melanosis will die before 10 years of age.^1^, ^3^

This aggressive entity found within the context of CMN is due to a different biological behavior with the presence of somatic mutations in 81% of cases in the NRAS gene of the melanocytes, in detriment of the mutations BRAF, demonstrating that they are genetically different from nevi developed after birth and an important risk factor for primary CNS and cutaneous melanoma.^4^

NRAS-mutant tumors tend to behave more aggressively particularly in early stages of the disease. In view of this differential genetic behavior, target therapies have been investigated for CNS melanoma in patients with CMN and the proven mutation of the protoncogene NRAS. Initial studies have demonstrated results of MEK inhibitors, Trametinib, in symptom control and improved quality of life, an important step in the discovery of treatment for this condition.^3^, ^5^

Evidence indicates a higher incidence of this neoplasm in patients presenting multiple satellite lesions, such as the pattern in garment-like, and/or paravertebral or axial location.^1^, ^3^

CNS melanoma currently emerges as the major limiting prognostic factor in children with CMN. In this scenario, cutaneous melanoma plays a less decisive role, influencing the decision toward prophylactic surgical excision. Brain MRI is important in this scenario, which should preferably be performed in the first year of life, since the incidence of CNS and cutaneous MM in the group with altered examination is 12%, as opposed to MM incidence of 1% in the group with normal CNS MRI at birth. The clinical follow-up of patients with altered MRI examinations should be annual.^3^

## Financial support

None declared.

## Authors’ contributions

Adriana Kamilly Leitão Pitman Machado: Approval of the final version of the manuscript; conception and planning of the study; elaboration and writing of the manuscript; critical review of the literature; critical review of the manuscript.

Débora Bacellar Cruz Nunes: Elaboration and writing of the manuscript.

Francisca Regina Oliveira Carneiro: Intellectual participation in the propaedeutic and/or therapeutic conduct of the studied cases.

Alena Margareth Darwich Mendes: Intellectual participation in the propaedeutic and/or therapeutic conduct of the studied cases.

## Conflicts of interest

None declared.
